# The Impact of Prolonged Stress of COVID-19 Pandemic and Earthquakes on Internet-Based Addictive Behaviour and Quality of Life in Croatia

**DOI:** 10.3390/ijerph22101587

**Published:** 2025-10-19

**Authors:** Zrnka Kovačić Petrović, Tina Peraica, Mirta Blažev, Dragica Kozarić-Kovačić

**Affiliations:** 1 Department of Psychiatry and Psychological Medicine, School of Medicine, University of Zagreb, 10000 Zagreb, Croatia; zrnka.kovacic@gmail.com; 2 Department of Addiction, University Hospital Vrapče, 10000 Zagreb, Croatia; 3 Department of Psychiatry, Referral Center for Stress-Related Disorders of the Ministry of Health, University Hospital Dubrava, 10000 Zagreb, Croatia; 4 Department of Forensic Sciences, University of Split, 21000 Split, Croatia; dkozaric_kovacic@yahoo.com; 5 Ivo Pilar Institute of Social Sciences, 10000 Zagreb, Croatia; blazevm@yahoo.com

**Keywords:** Internet-based addictive behaviour, quality of life, COVID-19 pandemic and earthquakes, stress, anxiety, depression, structural equation modelling approach

## Abstract

Prolonged stress caused by the COVID-19 pandemic and two concurrent earthquakes in 2020 increased Internet-based addictive behaviour, leading to decrease in mental health and quality of life (QoL) in the adult Croatian population. This study examined the association between Internet-based addictive behaviour and QoL during prolonged stress (pandemic and earthquakes). Specifically, it explored direct associations between QoL domains and overall/specific Internet use, problematic Internet use (PIU), and symptoms of anxiety, depression, and stress, as well as the indirect role of these symptoms in mediating the relationship between PIU and QoL. A cross-sectional online survey was conducted in autumn 2021 with a convenience sample (N = 1004; 82.2% women; M age = 34.98, SD = 12.24). Measures included increased overall and specific Internet use, PIU, stress (Impact of Event Scale), anxiety and depression symptoms (Hospital Anxiety and Depression Scale), and QoL (WHOQoL-BREF). Structural equation modelling showed that increased Internet use and PIU were directly associated with more severe symptoms of depression, anxiety, and stress, and with lower QoL. Significant indirect effects were also found: higher PIU, social media use, online shopping, and pornography viewing predicted greater depression, anxiety, and stress symptoms, which in turn predicted reduced QoL across multiple domains. These findings suggest that problematic and increased Internet use during periods associated with prolonged stress contribute to lower QoL through elevated psychological distress.

## 1. Introduction

The COVID-19 pandemic induced stress, anxiety, and depression [1,2], leading to a decreased quality of life (QoL) [3]. Quarantine has had additional, wide-ranging, and long-lasting psychological effects [4]. Natural disasters, like earthquakes, have also been shown to impact mental health by causing depression, post-traumatic stress disorder (PTSD), and functional disabilities [5,6], with short- and long-term effects on QoL outcomes [7] and decreased QoL [8,9]. Adverse situations like these generally elicit complex psychosocial responses that affect health-related QoL and need to be considered in biological, psychological, social, and economic terms [10,11,12,13,14].

According to the International Classification of Disease (ICD-11) and the Diagnostic and Statistical Manual of Mental Disorders (DSM V), individual interests in relation to Internet content can be divided into gambling, gaming, social media use, pornography use, or shopping [15,16]. Problematic Internet use (PIU) is considered a behavioural addiction, and this umbrella term covers a wide range of problematic online behaviours including excessive social media use, gaming, gambling, streaming, pornography viewing, and impulsive buying [17], as well as newer behaviours such as cyberhoarding and cyberchondria [18]. Researchers have used this term to describe the same problem: “individuals having trouble limiting their use of Internet to such an extent that their use has negative consequences” [19].

The prevalence of Internet-based addictive behaviours during the COVID-19 pandemic has increased worldwide [14,20]. An increase in PIU has been found in Croatia as well [21]. There is an association between PIU and QoL, but depression, anxiety, and stress appear to be strong mediators of this relationship [22]. Although frequent use of social media after the COVID-19 outbreak seems to be associated with poorer mental and psychosocial health in persons experiencing a high level of emotional distress and loneliness [23], some studies have reported different findings. One study found a positive relationship between COVID-19-related QoL and loneliness, with loneliness positively predicting Internet addiction, and a positive relationship between loneliness and happiness, i.e., as the loneliness of individuals increased, so did their level of happiness [24]. Furthermore, potential benefits of Internet use were found among middle-aged and older people (aged 55–75) who used the Internet for communication purposes; they had higher QoL [25].

During the first three waves of the COVID-19 pandemic (first: from mid-March to early May 2020; second: from the end of September to mid-February 2021; third: from mid-February to early June 2021), Croatia was hit by two earthquakes, one in Zagreb (capital city) on 22 March 2020, and the other in Petrinja (a town approximately 80 km SE from Zagreb) on 29 December 2020, both of which affected a large part of the population in Croatia.

With respect to the relationship between QoL and Internet-based addictive behaviour in populations that experienced earthquakes, our literature search did not identify any studies. To the best of our knowledge, this is the first study to examine the relationship between QoL and overall and specific Internet activity use, and PIU, among adults who have experienced the COVID-19 pandemic and earthquakes at the same time.

This study is an extension of our earlier research on the relationship between the COVID-19 pandemic and earthquakes and problematic Internet behaviours [21], and the impact of the first three COVID-19 pandemic waves and two earthquakes on the QoL of the general adult population in Croatia [26]. However, the current study presents a distinct analytical focus, conceptual framework, and statistical approach, including variables and models not covered in earlier publications. In our previous study [21], we provided descriptive and correlational analyses of problematic Internet-based addictive behaviour and its association with symptoms of anxiety, depression, and stress before and during prolonged stress periods caused by the COVID-19 pandemic and earthquakes. In another study [26], we explored a broader set of predictors of various QoL domains and conducted a series of regression analyses to examine associations between five blocks of predictors (sociodemographic characteristics, COVID-19-related stressors, earthquake-related stressors, perceived stress, anxiety, and depression symptoms) and QoL domains in the adult population. In this study, we applied structural equation modelling to simultaneously evaluate the mediating effects of psychological symptoms on the relationship between Internet-based addictive behaviour and QoL domains. This integrative approach extends beyond prior analyses by linking Internet-based addictive behaviour, psychological symptoms, and QoL variables in a single, empirically tested model within the unique context of experiencing two concurrent large-scale stressors.

Our hypothesis was that lower QoL scores were directly associated with an increase in Internet-based addictive behaviour (overall and specific Internet use), PIU, and symptoms of stress, anxiety, and depression. Furthermore, we hypothesised that higher levels of PIU would indirectly predict poorer QoL across various domains through more severe symptoms of stress, anxiety, and depression.

The first aim of the study was to determine the direct association between different QoL domains and (a) an increase in overall and specific Internet activity use, (b) PIU during prolonged stress caused by the COVID-19 pandemic and earthquakes, and (c) stress, anxiety, and depression symptoms. The second aim was to determine how an increase in the overall and specific Internet activity use and PIU during prolonged stress predicted different QoL domains indirectly, i.e., through stress, anxiety, and depression symptoms.

## 2. Materials and Methods

### 2.1. Study Design

This is an Internet-based quantitative, cross-sectional study with a non-probabilistic convenience sample, meaning participants were recruited based on their accessibility and willingness to participate, rather than through random selection. Thus, the survey link was distributed via social networks, and all individuals who met the inclusion criteria and chose to respond were included in the sample.

### 2.2. Participants

Of 1286 individuals from across Croatia who took the online survey, 282 were not included in the analysis. They submitted partially completed questionnaires or were excluded from the study according to the exclusion criteria. The final sample consisted of 1004 participants (82.2% women). Participants were at average 34.98 (*SD* = 12.24) years old, with completed undergraduate or graduate education (56%) and employed (61.8%) with an average of 10.27 (*SD* = 1.05) years of work experience. The majority of participants were in a relationship (25.3%) or married (30.7%), without children (64.9%), and from more urban areas in Croatia (>7000 inhabitants; 59.6%).

Participants were eligible for inclusion in the study if they were Croatian residents during the three pandemic waves and two earthquakes, aged 18 or over, and able to complete the online survey without difficulties. Not understanding written Croatian, not signing the informed consent, or missing data were criteria for exclusion. Participation was completely voluntary, and no financial incentive was offered.

### 2.3. Method

The authors created an online survey and sent the survey hyperlink to the recipients through Facebook, Twitter, LinkedIn, Google+, and Instagram. The aims of the study and participants’ rights were described on the survey’s first page. The survey took about 20 min to complete and was available on a Google-built website.

Data were collected from September 30, 2021, to October 17, 2021, as part of a research project investigating the impact of the COVID-19 pandemic and earthquakes on mental health of the Croatian population. Only the methods relevant to the present analysis are described here, while a detailed description may be found in earlier published articles [21,26].

### 2.4. Measures

Increase in overall and specific Internet use (online gambling, online gaming, pornography viewing, social media, online shopping) during prolonged stress caused by the COVID-19 pandemic and earthquakes were measured using a dichotomous (no/yes) item: “Have you increased your overall and specific Internet use (online gambling; online gaming, pornography viewing, social media, online shopping) during prolonged stress (COVID-19 pandemic and earthquakes)?” separately for overall Internet use and each specific Internet activity. The total score for overall use and specific Internet activity was either 0—no increase or 1—increase in overall and specific Internet use during prolonged stress. Because only 12 participants reported online gambling, they were excluded from further analysis to avoid a non-meaningful inferential analysis [27].

PIU was measured using a symptom-based criteria list consisting of eight items, as proposed by Tao et al. (2010) [28]. To be categorised as PIU and problematic specific Internet activity use, a participant had to check two symptoms (preoccupation and withdrawal symptoms) and any of the other listed symptoms (1. tolerance, 2. lack of control, 3. continued excessive use despite the knowledge of negative effects/affects, 4. loss of interests other than for the Internet, 5. use of the Internet to escape or relieve a dysphoric mood, 6. hiding from friends, relatives, important relationships, or career opportunities due to Internet use, and duration of Internet use of at least 6 h daily for at least three months during the first three pandemic waves that overlapped with earthquakes). The total PIU score was then dichotomised and expressed separately, with 0 indicating non-PIU and 1 indicating PIU. Kuder–Richardson internal reliability [29] in our sample was 0.85.

Stress symptoms were measured by the Impact of Event Scale (IES) [30], a 15-item questionnaire evaluating experiences of avoidance and intrusion to “reflect the intensity of the post-traumatic phenomena” on a 4-point Likert scale (not at all = 0; rarely = 1; sometimes = 3; often = 5). The instructions for the IES were specifically tailored to match the context of the week before data collection. The total score ranges from 0 to 70, with higher scores indicating more severe stress symptoms. The intrusion subscale score ranges from 0 to 35, and the avoidance subscale score ranges from 0 to 40. Cronbach’s α in our sample was 0.92 for IES, 0.90 for IES-Intrusion, and 0.86 for IES-Avoidance. The reliability and validity of the IES were verified in previous studies [30,31]. Validation was originally performed by Horowitz et al. [30] and results indicated a test–retest reliability of 0.87 for the total stress score, 0.89 for the intrusion subscale, and 0.79 for the avoidance subscale. Both subscales showed satisfactory reliability in adult subjects (Cronbach’s α 0.88 and 0.89 for the intrusion and avoidance subscales, respectively) [31].

Anxiety and depression symptoms were evaluated using the Hospital Anxiety Depression Scale (HADS) [32], a 14-item screening tool for detecting the symptoms of anxiety (The Hospital Anxiety Depression Scale-Anxiety; HADS-A) and depression (The Hospital Anxiety Depression Scale-Depression; HADS-D) on a 3-point Likert scale from 0 to 3 (0 = not at all, 3 = all the time). Participants self-assessed their anxiety and depression symptoms experienced in the week before data collection. Higher scores indicate more severe anxiety and depression symptoms. Cronbach’s α in our sample was 0.909 for total HADS score, 0.89 for HADS-A subscale, and 0.83 for HADS-D subscale. In primary care patients and the general population, Cronbach’s α for HADS-A varied from 0.68 to 0.93 (mean 0.83) and for HADS-D from 0.67 to 0.90 (mean 0.82) [33]. Furthermore, results indicated a test–retest reliability of 0.89 for the total HADS score, 0.87 for the HADS-A, and 0.81 for the HADS-D subscale in a population of family caregivers of patients with Alzheimer’s disease in Croatia [34].

Quality of life was measured with the World Health Organisation Quality of Life (WHOQoL-BREF)—a 26-item, 5-point Likert scale questionnaire [35,36,37]—which is commonly used to measure general QoL, health satisfaction, physical and psychological health, social relationships, and environment. Higher scores indicate more positive perceptions of QoL. In the study, the WHOQoL-BREF scale instructions specifically referred to the week before data collection. The QoL scale’s reliability was as follows: physical health (α = 0.81), psychological health (α = 0.89), social relationships (α = 0.70), and environment (α = 0.78).

### 2.5. Ethical Procedures

This study obtained approval from the Ethics Committee of the University Hospital Vrapče in Zagreb (Institutional Review Board approval date: 31 May 2021; reference number: 23-1064/3-21). All participants included in the study provided informed consent in accordance with ethical principles in human research (only those who provided their consent were allowed to complete the survey).

### 2.6. Data Analysis

**Figure 1:**
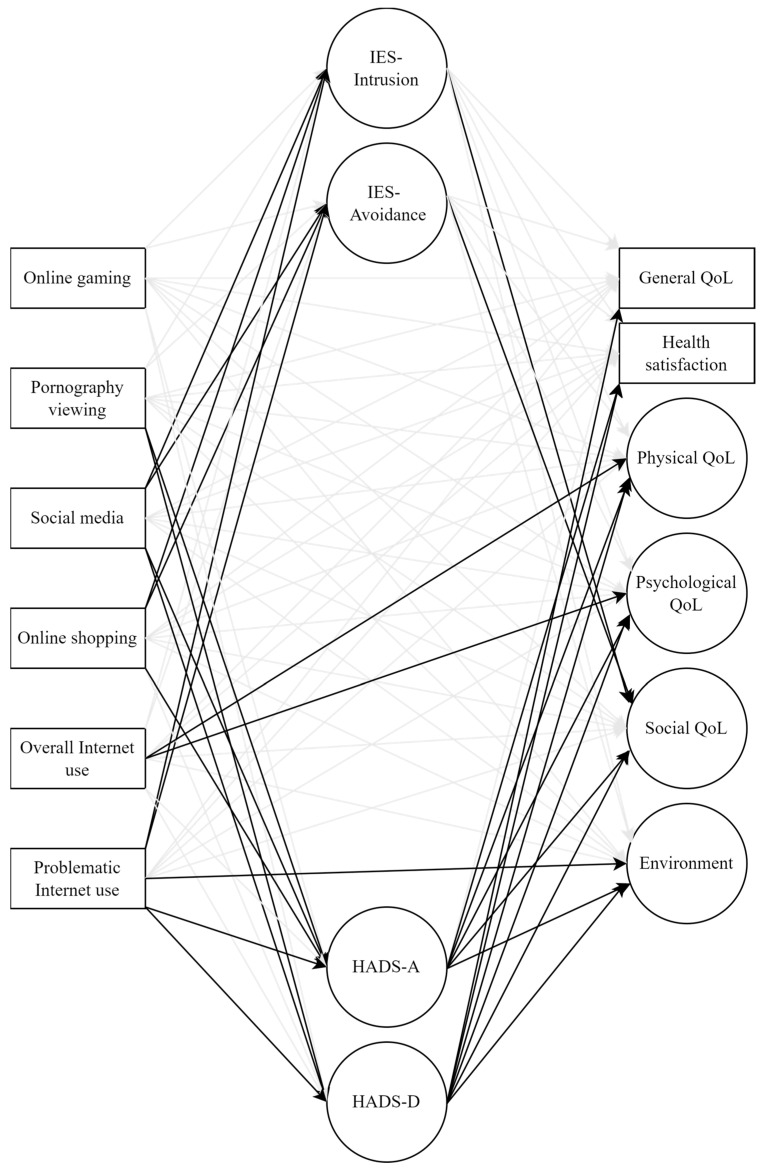
Structural equation modelling: tested model. Note. Indicators for latent variables, as well as errors and residuals were omitted from the figure due to clarity; black arrows represent regression paths that are significant at *p* < 0.05, while grey arrows represent tested, but non-significant regression paths.

To verify the assumption of normality, Kolmogorov–Smirnov test was calculated for all quantitative variables, i.e., QoL domains and stress, anxiety, and depression symptoms. To determine how overall and specific Internet use and PIU predict different QoL domains directly and indirectly through stress, anxiety, and depression symptoms, structural equation modelling was performed. The exogenous variables in the model were overall and specific Internet use and PIU (Figure 1). The mediators in the model were IES-Intrusion, IES-Avoidance, HADS-A, and HADS-D symptoms, while the endogenous variables in the model were general QoL, health satisfaction, physical and psychological health, social relationships, and environment. Full information maximum likelihood (FIML) method for handling all missing values (due to incomplete answers) was implemented with weighted least square mean and variance adjusted (WLSMV) estimator, which is known for good performance in conditions where a tested model is rather large, variables are categorical or ordinal, and considered a robust estimator in case of non-normal data distribution [38,39]. To evaluate model–data fit, the rules of thumb proposed by Hu and Bentler’s [40] and Browne and Cudeck’s [41] were adopted, i.e., a good model–data fit was considered good when chi-square *p* value > 0.05; chi-square and degrees of freedom ratio < 3 (good)/ < 5 (acceptable); the comparative fit index (CFI) and the Tucker–Lewis index (TLI) > 0.95 (good)/ > 0.90 (acceptable); root mean square error of approximation (RMSEA) and standardised root mean square residual (SRMR) < 0.08 (good)/ < 0.10 (acceptable); and p_close_ > 0.05 as good model–data fit. To quantify the magnitude of effects in the structural equation model, standardised beta coefficients (β) and the proportion of explained variance (*R*^2^) for each endogenous variable are reported as primary effect size measures, accompanied by 95% confidence intervals for all direct and indirect effects to indicate precision and statistical significance. Statistical power is supported by the large sample size (*N* = 1004), which exceeds recommended guidelines for models of this complexity, ensuring adequate sensitivity to detect meaningful effects [42]. The analyses were conducted using Jamovi (version 2.5.5) (https://www.jamovi.org/) (accessed on 28 July 2025).

## 3. Results

### 3.1. Descriptive Statistics and Normality Assumption Check

The participants mostly perceived their QoL as good, while reporting low to moderate stress, anxiety, and depression symptoms (Table 1). Also, around half of the participants increased their Internet use, while one quarter demonstrated PIU during prolonged stress.

Kolmogorov–Smirnov test indicated that data were not univariately normal for the QoL domain and stress, anxiety, and depression symptoms scales (*p* < 0.001, Table 1). However, because all skewness and kurtosis coefficients were around 0.5, we assumed that violations of the normality assumption were not too severe [42].

### 3.2. Structural Equation Modelling Results

The structural equation modelling results showed a relatively good model–data fit by Hu and Bentler’s [40] and Browne and Cudeck’s [41] criteria (Table 2). The model, with Internet use behaviours as exogenous variables and stress, anxiety, and depression symptoms as mediators (Figure 1), significantly explained 41.4% (*p* < 0.001) of general QoL, 30.9% (*p* < 0.001) of health satisfaction variance, and 65.8% (*p* < 0.001) of physical, 77.6% (*p* < 0.001) of psychological, 54.1% (*p* < 0.001) of social relationships, and 36.6% (*p* < 0.001) of environment domains.

#### 3.2.1. Direct Effects of Overall and Specific Internet Use and PIU During Prolonged Stress on QoL Domains

Increase in all specific Internet activities (online gaming, pornography viewing, social media consumption, and online shopping) were not directly associated with any QoL domain during prolonged stress (*p* > 0.05; Table 3). However, an increase in overall Internet use predicted poorer physical and psychological health, while higher PIU predicted lower environment satisfaction.

Also, more severe intrusion symptoms predicted better social relationships, whereas more severe avoidance symptoms predicted poorer social relationships. Furthermore, increased anxiety and depression symptom severity predicted poorer outcomes across most QoL domains. All predictors, standardised regression coefficients, *p*-values, and 95% confidence intervals are detailed in Table 3.

#### 3.2.2. Direct Effects of Overall and Specific Internet Use, and PIU During Prolonged Stress on Stress, Anxiety, and Depression Symptoms

Increase in social media consumption, online shopping, and PIU during prolonged stress predicted stronger intrusion and avoidance stress symptoms (Table 4). Additionally, increases in pornography viewing, social media consumption, and PIU predicted more severe anxiety and depression symptoms, whereas increases in online shopping predicted only greater anxiety. The model did not show any significant predictive effects for online gaming or overall Internet use on stress, anxiety, or depression outcomes (*p* > 0.05). Complete regression statistics are available in Table 4.

#### 3.2.3. Indirect Effects of Overall and Specific Internet Use and PIU During Prolonged Stress on QoL Domains Through Intrusion and Avoidance Stress Symptoms

Increased social media use (β = 0.04, 95% CI [0.00, 0.07], *p* = 0.048), online shopping (β = 0.03, 95% CI [0.01, 0.05], *p* = 0.011), and problematic Internet use (PIU) (β = 0.02, 95% CI [0.00, 0.04], *p* = 0.026) were associated with better social relationships through greater intrusion stress symptoms (Table 5). Conversely, only online shopping showed a small but significant negative indirect effect on social relationships via avoidance stress symptoms (β = −0.02, 95% CI [−0.03, 0.00], *p* = 0.047). No other significant indirect effects were observed through these stress pathways.

#### 3.2.4. Indirect Effects of Overall and Specific Internet Use, and PIU During Prolonged Stress on QoL Domains Through Anxiety and Depression Symptoms

Increases in pornography viewing predicted elevated anxiety symptoms, which in turn were linked to worse physical (β = −0.03, 95% CI [−0.05, −0.01], *p* = 0.014), psychological (β = −0.02, 95% CI [−0.04, −0.01], *p* = 0.013), and environmental QoL (β = −0.03, 95% CI [−0.05, −0.00], *p* = 0.024). Similarly, online shopping was related to increased anxiety mediating poorer physical (β = −0.02, 95% CI [−0.05, −0.00], *p* = 0.042) and psychological health (β = −0.02, 95% CI [−0.04, −0.00], *p* = 0.041). PIU demonstrated broader negative indirect associations through anxiety with health satisfaction, physical, psychological, social, and environmental domains (βs ranging −0.03 to −0.04, all *p* < 0.05).

Additionally, pornography viewing, social media use, and PIU predicted elevated depression symptoms, which in turn significantly predicted worse outcomes across all QoL domains (βs from −0.02 to −0.14, all *p* < 0.05).

## 4. Discussion

In the present study, we found that a quarter of our participants reported PIU and around half of them increased overall Internet use, which is in line with our previous findings [21], a systematic review, which included studies from other countries [20], as well as a narrative review [14]. Our study showed direct effects of overall and specific Internet activities and PIU during prolonged stress on QoL domains and stress, anxiety, and depression symptoms. Furthermore, indirect effects of overall and specific Internet activities and PIU during prolonged stress on QoL domains through intrusion and avoidance stress symptoms, anxiety, and depression symptoms were found.

The model, with Internet use behaviours as exogenous variables and stress, anxiety, and depression symptoms as mediators, significantly explained three quarters of psychological health, more than half of physical health, around half of social relationships and general QoL, and around one third of environment and health satisfaction variance. It is important to assess both global QoL domains (general QoL and health satisfaction) and specific QoL domains in order to have a broader insight into different areas of QoL and to be able to undertake preventative activities and offer possible treatment [43,44]. This study shares several similarities with studies conducted in other countries during the COVID-19 pandemic, although samples and age groups differed [10,11,12,13,14,22,25,45].

Our findings indicate that overall and Internet-specific activities and PIU were directly associated with QoL among adults via stress, anxiety, and depression. In other words, Internet-based addictive behaviour had a strong positive association with stress, anxiety, and depression, which then negatively influenced the participants’ QoL and possibly contributed to psychological distress [46]. Although it could be expected that stress symptoms negatively affect social relationships, a possible explanation of our findings is that avoidance stress symptoms limit people in their social relationships, which consequently leads to reduced social relationships. On the other hand, intrusive stress symptoms improve social relationships due to the avoidance of intrusive thoughts and their externalisation through social relationships. These findings are consistent with previous studies that found the impact of stress, anxiety, and depression symptoms on QoL in prolonged stress situations [1,2,26,47,48,49,50].

Increases in online gaming, pornography viewing, social media use, and online shopping during prolonged stress did not significantly predict any of the QoL domains directly. Our findings that an increase in social media use predicted stronger intrusion and avoidance symptoms and more severe anxiety and depression symptoms are in line with a cross-country comparative survey that suggested that social media use after the COVID-19 outbreak was associated with poorer mental and psychosocial health in those who experienced high levels of emotional distress and loneliness [23]. The same applied to PIU. One study found that negative feelings and perceived stress during both work-time and leisure-time Internet use can be risk factors for mental health in terms of PIU and perceived lower QoL [51]. At the same time, increases in online gaming and overall Internet use did not significantly predict stress, anxiety, and depression symptoms. This is opposite to a study carried out among adolescents, which showed that depression, anxiety, and stress served as a strong mediator in the association between Internet gaming disorder, insomnia, and QoL [22]. We found no similar studies to compare our results regarding increased online shopping and pornography viewing in the prediction of stress, anxiety, and depression symptoms. In our study, we applied WHOQoL-BREF [35,36,37], which, in a systematic review, proved to be the most relevant instrument for adults among the existing instruments of QoL, although new measurement instruments are needed to target QoL domains specifically relevant in the context of PIU [52].

Additionally, we found that overall and Internet-specific activities use and PIU were indirectly associated with QoL among adults via stress, anxiety, and depression. Our study confirm that psychological distress, anxiety, and depression are mediators in the association between PIU and Internet-based addictive behaviour and QoL. Most research published in the last three years has shown that PIU, or Internet-based addictive behaviour, has become an internationally relevant public health issue [53]. A significantly negative relationship was shown between PIU and the psychological and physical health domains [52], especially during the pandemic [20], while the findings regarding the environment and social relationships were inconsistent due to the heterogeneity of instruments used [20].

The practical implications of our findings show the need to raise awareness about the risks of Internet-based addictive behaviour and decreased QoL during and after prolonged stress (pandemic and earthquakes). Policymakers and clinicians should reduce the negative impact of prolonged stress on Internet-based addictive behaviour and QoL by implementing prevention and treatment interventions for vulnerable groups to reduce stress, anxiety, and depression.

Although this study provides significant findings regarding the relationships between prolonged stress (COVID-19 and earthquakes), QoL, stress, anxiety, and depression, and Internet-based addictive behaviour, it undoubtedly has certain limitations. The cross-sectional design of the study provided only strong associations between variables of interest. To examine causality effects and other parameters of Internet-based addictive behaviour impact on adult QoL, a longitudinal study or randomised control trial should be performed. Because this study was carried out among an adult population, the findings may not be generalisable to children and adolescents. Further limitations of the study are the same as those presented in our previous articles [21,26]. Although we used structural equation modelling for data analysis as a more robust method, caution should be exercised when interpreting the mediation findings due to the cross-sectional design.

Future research dimensions should be longitudinal and focus on individuals with PIU and reduced QoL who experience greater stress and mental health symptoms in situations of prolonged stress. Further prospective studies focusing on specific types of stressors (pandemic/earthquakes) on recreational Internet use, PIU, and QoL during and after prolonged stress are also needed.

## 5. Conclusions

In conclusion, according to structural equation modelling, the current study proposed a new model for examining the relationships between the impact of prolonged stress (COVID-19 pandemic and earthquakes), QoL, PIU, increased overall and specific Internet activities, and stress, anxiety, and depression symptoms among adults. The findings revealed that the impact of prolonged stress on the QoL has a direct or indirect effect on stress, anxiety, depression, and PIU, as well as an increase in overall and specific Internet activities among adults. The model showed a direct association between Internet-based addictive behaviour and QoL, as well as between increases in overall and specific Internet activities and PIU, and stress, anxiety, and depression. It is even more important that there was an indirect association between Internet-based addictive behaviour and QoL through stress, anxiety, and depression, which led to a reduction in the QoL.

## Figures and Tables

**Table 1:** Descriptive statistics and Kolmogorov–Smirnov test of normality (N = 1004).

Variables	*M (SD)*	*Min*	*Max*	*Skew.*	*Kurt.*	*K–S*
General QoL	3.81 (0.92)	1	5	−0.44	−0.23	0.23 *
Health satisfaction	3.88 (0.99)	1	5	−0.68	−0.04	0.23 *
Physical health	15.77 (3.03)	4.57	20	−0.77	0.33	0.10 *
Psychological health	14.63 (3.50)	4	20	−0.56	−0.38	0.10 *
Social relationships	14.89 (3.69)	4	20	−0.47	−0.35	0.10 *
Environment	15.71 (2.71)	5.50	20	−0.72	0.44	0.08 *
IES-Intrusion	12.44 (9.98)	0	35	0.62	−0.67	0.12 *
IES-Avoidance	11.52 (9.37)	0	40	0.76	−0.08	0.11 *
HADS-Anxiety	8.31 (4.78)	0	21	0.39	−0.44	0.08 *
HADS-Depression	5.90 (4.38)	0	21	0.76	0.04	0.12 *
Increase in use	*n* (%)					
Online gaming	118 (10.6)					
Pornography viewing	71 (6.4)					
Social media	540 (48.3)					
Online shopping	307 (27.5)					
Overall Internet use	586 (52.4)					
Problematic Internet use	264 (26.3)					

Note. Skew.—Skewness; Kurt.—Kurtosis; K–S: Kolmogorov–Smirnov normality test; * *p* < 0.001.

**Table 2:** Model fit indices for structural equation modelling of the tested model.

Fit Index	Model Value	Recommended Cutoff	Interpretation
χ^2^	3931.1 *	*p* > 0.05	Bad fit
χ^2^/*df*	2.76	<3.00 (good), <5.00 (acceptable)	Good fit
CFI	0.910	>0.95 (good), >0.90 (acceptable)	Acceptable fit
TLI	0.900	>0.95 (good), >0.90 (acceptable)	Acceptable fit
RMSEA	0.042	<0.05 (good), <0.08 (acceptable)	Good fit
90% CI RMSEA	0.040, 0.043	The upper bound ≤ 0.05	Good fit
*p*_close_	0.999	*p* > 0.05	Good fit
SRMR	0.073	<0.08 (good)	Good fit

Note. * *p* < 0.001.

**Table 3:** Direct effects of overall and specific Internet use and problematic Internet use during prolonged stress on QoL domains (N = 1004).

	General QoL			Health Satisfaction			Physical Health		
Direct effects	β	*p*	95% CI	β	*p*	95% CI	β	*p*	95% CI
Online gaming	0.00	0.957	−0.06, 0.05	−0.02	0.552	−0.08, 0.04	0.00	0.997	−0.05, 0.05
Pornography viewing	−0.04	0.155	−0.09, 0.02	−0.03	0.350	−0.08, 0.03	0.02	0.511	−0.03, 0.07
Social media	0.04	0.462	−0.07, 0.15	−0.03	0.633	−0.15, 0.09	0.09	0.104	−0.02, 0.20
Online shopping	0.04	0.191	−0.02, 0.10	0.05	0.114	−0.01, 0.12	0.03	0.382	−0.03, 0.08
Internet use	−0.06	0.279	−0.18, 0.05	−0.01	0.906	−0.13, 0.12	−0.19 **	0.001	−0.30, −0.08
Problematic Internet use	−0.03	0.226	−0.09, 0.02	0.01	0.760	−0.05, 0.06	−0.04	0.147	−0.09, 0.01
IES-Intrusion	−0.03	0.572	−0.13, 0.07	0.02	0.665	−0.09, 0.14	−0.02	0.694	−0.12, 0.08
IES-Avoidance	0.01	0.874	−0.09, 0.10	−0.05	0.295	−0.15, 0.05	−0.04	0.381	−0.13, 0.05
HADS-Anxiety	−0.06	0.296	−0.17, 0.05	−0.16 **	0.008	−0.28, −0.04	−0.27 **	<0.001	−0.38, −0.16
HADS-Depression	−0.58 **	<0.001	−0.68, −0.48	−0.41 **	<0.001	−0.52, −0.31	−0.54 **	<0.001	−0.64, −0.45
	**Psychological Health**			**Social** **Relationships**			**Environment**		
Direct effects	β	*p*	95% CI	β	*p*	95% CI	β	*p*	95% CI
Online gaming	0.02	0.435	−0.03, 0.06	−0.04	0.264	−0.01, 0.03	−0.04	0.210	−0.11, 0.02
Pornography viewing	−0.04	0.108	−0.08, 0.01	−0.05	0.090	−0.11, 0.01	−0.02	0.538	−0.09, 0.05
Social media	0.07	0.103	−0.02, 0.16	0.03	0.603	−0.09, 0.16	−0.11	0.111	−0.25, 0.03
Online shopping	0.02	0.416	−0.03, 0.07	0.00	0.946	−0.07, 0.07	−0.01	0.748	−0.09, 0.06
Internet use	−0.10 *	0.038	−0.19, −0.01	−0.08	0.254	−0.21, 0.06	0.05	0.534	−0.10, 0.19
Problematic Internet use	−0.01	0.636	−0.05, 0.03	0.03	0.373	−0.03, 0.09	−0.07 *	0.037	−0.13, −0.00
IES-Intrusion	0.07	0.112	−0.02, 0.15	0.21 **	<0.001	0.10, 0.33	0.04	0.597	−0.09, 0.16
IES-Avoidance	0.01	0.843	−0.07, 0.08	−0.14 **	0.008	−0.25, −0.04	−0.06	0.345	−0.17, 0.06
HADS-Anxiety	−0.24 **	<0.001	−0.33, −0.15	−0.16 *	0.018	−0.29, −0.03	−0.27 **	<0.001	−0.41, −0.13
HADS-Depression	−0.69 **	<0.001	−0.77, −0.61	−0.59 **	<0.001	−0.70, −0.48	−0.32 **	<0.001	−0.44, −0.19

Note. QoL—Quality of life; IES—Impact Event Scale; HADS—Hospital Anxiety Depression Scale; * *p* < 0.05; ** *p* < 0.01.

**Table 4:** Direct effects of overall and specific Internet use and problematic Internet use during prolonged stress on stress, anxiety, and depression symptoms (N = 1004).

	IES-Intrusion			IES-Avoidance		
Direct effects	β	*p*	95% CI	β	*p*	95% CI
Online gaming	0.05	0.177	−0.02, 0.11	0.04	0.278	−0.03, 0.11
Pornography viewing	−0.01	0.692	−0.08, 0.05	0.00	0.937	−0.07, 07
Social media	0.17 *	0.017	0.03, 0.31	0.21 **	0.004	0.07, 0.35
Online shopping	0.14 **	<0.001	0.06, 0.21	0.12 **	0.003	0.04, 0.20
Internet use	0.01	0.881	−0.13, 0.16	−0.03	0.679	−0.18, 0.12
Problematic Internet use	0.09 **	0.004	0.03, 0.16	0.10 **	0.004	0.03, 0.17
	**HADS−Anxiety**			**HADS−Depression**		
Direct effects	β	*p*	95% CI	β	*p*	95% CI
Online gaming	0.03	0.376	−0.04, 0.10	0.07	0.052	−0.00, 0.14
Pornography viewing	0.10 **	0.004	0.03, 0.16	0.07 *	0.032	0.01, 0.14
Social media	0.14 *	0.047	0.00, 0.28	0.20 **	0.005	0.06, 0.34
Online shopping	0.09 *	0.025	0.01, 0.17	0.01	0.727	−0.06, 0.09
Internet use	0.05	0.507	−0.10, 0.20	0.00	0.988	−0.15, 0.15
Problematic Internet use	0.16 **	<0.001	0.09, 0.22	0.13 **	<0.001	0.07, 0.20

Note. IES—Impact Event Scale; HADS—Hospital Anxiety Depression Scale; * *p* < 0.05; ** *p* < 0.01.

**Table 5:** Significant indirect effects of overall and specific Internet use and problematic Internet use during prolonged stress on QoL domains via stress, anxiety, and depression (N = 1004).

Predictor	Mediator	QoL Domain(s) Affected
Online gambling	n.s.	n.s.
Pornography viewing	Anxiety	Physical health, psychological health, and environment
	Depression	All domains
Social media use	Intrusion	Social relationships
	Depression	All domains
Online shopping	Intrusion	Social relationships
	Avoidance	Social relationships
	Anxiety	Physical health and psychological health
Overall Internet use	n.s.	n.s.
Problematic Internet use	Intrusion	Social relationships
	Anxiety	Health satisfaction, physical health, psychological health, social relationships, and environment
	Depression	All domains

Note. QoL—Quality of life; n.s.—non-significant effect. Only statistically significant indirect effects (*p* < 0.05) are displayed.

## Data Availability

The register-based data that support the findings of this study are available on request from the corresponding author.
